# Erk1/2 Orchestrates SSPH I‐Induced Oxidative Stress, Mitochondrial Dysfunction and Ferroptosis in Hepatocellular Carcinoma

**DOI:** 10.1111/jcmm.70609

**Published:** 2025-05-20

**Authors:** Yuewen Sun, Ying Zhou, Dan Huang, Zhiguang Zhao, Qingrui Shao, Jianzhe Li, Xiaofang Zhao, Xudong Liu

**Affiliations:** ^1^ Guangxi University of Chinese Medicine Nanning China; ^2^ Guangxi Vocational University of Agriculture Nanning China; ^3^ Yunnan Grassroots Medical Industry Development Co. Ltd Kunming China

**Keywords:** Erk1/2, ferroptosis, Nrf1/2, ROS, SSPH I

## Abstract

Although Erk1/2 has been linked to oxidative stress regulation in hepatocellular carcinoma (HCC), the interplay among Erk1/2, reactive oxygen species (ROS), and iron metabolism remains poorly characterised. The steroidal saponin SSPH I, a recognised ferroptosis inducer, exerts dual pharmacological effects via Erk1/2 and ROS‐dependent pathways. This study aimed to investigate the regulatory mechanisms of Erk1/2 in ferroptosis and oxidative stress and analyse their feedback regulatory effects on Erk1/2 in HCC using SSPH I as a pharmacological probe, and further elucidate the anti‐HCC effects and mechanisms of SSPH I in vitro and in vivo. Mechanistic studies utilised three inhibitors: U0126 (Erk1/2 phosphorylation inhibitor), Ferrostatin‐1 (ferroptosis inhibitor), and N‐acetyl cysteine (ROS scavenger), combined with SSPH I to delineate its effects on cell viability, mitochondrial dynamics, ferroptosis induction and oxidative stress. Mechanistically, SSPH I disrupted mitochondrial function and suppressed HCC cell survival through iron accumulation and ROS generation, while concurrently activating Erk1/2 signalling. Pharmacological inhibition of ROS or iron pathways partially attenuated SSPH I‐induced ferroptosis and ROS generation, but failed to abrogate these effects. Erk1/2 inhibition completely abolished SSPH I‐mediated regulation of the Nrf1/2‐HO‐1 axis and ferroptosis‐related protein expression in cellular and animal models, identifying Erk1/2 as the upstream regulatory node. Notably, while both SSPH I and U0126 monotherapies inhibited xenograft growth, their combined use resulted in antagonistic effects. These findings establish Erk1/2 activation as the central molecular mechanism orchestrating SSPH I‐driven oxidative stress amplification, mitochondrial dysfunction and ferroptosis execution in HCC.

## 1 Introduction

Hepatocellular carcinoma (HCC) is one of the leading causes of cancer‐related death factors worldwide, with its mortality rate continuing to rise [1]. The MAPK/ERK signalling pathway not only plays a pivotal role in HCC carcinogenesis, progression and treatment but is also a target of first‐line therapies such as Sorafenib and Lenvatinib [2]. Constitutively activated Erk1/2 is frequently observed in HCC patients and correlates with poor prognosis [3, 4].

Erk1/2‐mediated oxidative stress regulation represents a critical mechanism in HCC pathogenesis. Specifically, Erk1/2 drives mitochondrial ROS production, thereby facilitating tumour cell migration and proliferation [5]. Paradoxically, Erk1/2 activation simultaneously mitigates ROS‐induced damage through modulation of mitochondrial homeostasis [6, 7]. This dual functionality appears orchestrated through interactions with Nrf2, as emerging evidence suggests the Erk1/2‐Nrf2 axis confers protection against oxidative stress, mitochondrial dysfunction and ferroptosis in hepatic tissues [8, 9].

Despite established connections between Erk1/2 and ferroptosis/oxidative stress in HCC, three critical knowledge gaps persist. First, the temporal hierarchy within the Erk1/2‐ferric ion‐ROS regulatory loop remains elusive. While some studies propose that ROS accumulation initiates Erk1/2 activation [6, 10], contrary evidence demonstrates ROS‐mediated feedback inhibition of Erk1/2 [11]. Notably, Erk1/2 hyperphosphorylation itself may serve as the instigating event in oxidative damage preceding cirrhosis [11, 12]. Second, the context‐dependent role of Erk1/2 in ferroptosis regulation generates controversy: Under Nrf2‐deficient conditions, Erk1/2 activation elevates intracellular iron levels to potentiate ferroptosis [13, 14], whereas other models demonstrate that Erk1/2 inhibition triggers ferroptosis through ROS accumulation [15]. These discrepancies arise from the failure of prior studies to account for the reciprocal regulatory circuit connecting Erk1/2, ROS and ferroptosis, having only partially validated pairwise associations (e.g., Erk1/2‐ROS or Erk1/2‐ferroptosis). Furthermore, concurrent processes such as apoptosis, autophagy induction and system $X_{c}^{-\cdot }$‐inhibition confound mechanistic interpretation by introducing crosstalk within the Erk1/2‐ROS‐ferroptosis axis. It is necessary to comprehensively explain the regulatory role of Erk1/2 on oxidative stress and ferroptosis in HCC.

SSPH I (CAS: 290809‐72‐2), a bioactive steroidal saponin isolated from *Schizocapsa plantaginea Hance*, has been incorporated into traditional anticancer formulations. Our prior work identified SSPH I as a unique ferroptosis inducer in HCC that provokes iron accumulation and mitochondrial alterations characteristic of ferroptosis independent of system $X_{c}^{-\cdot }$‐inhibition [16]. While the published study demonstrated that Erk1/2 is correlated with SSPH I‐induced ROS elevation and autophagy inhibition in HCC cells, it crucially omitted investigation of the regulatory interplay between Erk1/2 and ROS in ferroptosis induction and failed to address the therapeutic implications of this mechanism for SSPH I's anti‐HCC efficacy [17]. Capitalising on the tripartite pharmacological profile of SSPH I, this study aims to elucidate the regulatory mechanisms of Erk1/2 in ferroptosis and oxidative stress pathways and analyse their feedback regulatory effects on Erk1/2 while systematically investigating the anti‐hepatocellular carcinoma efficacy and underlying mechanisms of SSPH I through integrated in vitro and in vivo approaches. The findings are expected to establish a theoretical foundation for refining Erk1/2‐directed therapeutic strategies in HCC management.

## 2 Materials and Methods

### 2.1 Materials and Reagents

The human HCC cell lines HepG2 and SMMC‐7721 were purchased from the Type Culture Collection of the Chinese Academy of Sciences, Shanghai, China. All cells used were at passages 4–8. SSPH I (purity: 95.68%) was isolated from the underground cane of *Schizocapsa plantaginea Hance* and purified using the procedures described in prior studies [16, 18, 19]. Primary antibodies Erk1/2 (ab184699), p‐Erk1/2 (ab278538), Nrf1 (ab175932), Nrf2 (ab62352), HO‐1 (ab68477), TFR (ab109259), ferroportin1 (fpn‐1, ab239583), SLC7A11 (ab307601), ferritin (ab75973), GAPDH (ab8245) and β‐actin (ab8226) were purchased from Abcam, Shanghai, China.

### 2.2 Cell Culture and Treatment

HCC cells were cultured at 37°C in 5% CO_2_ using high‐glucose Dulbecco's modified Eagle medium (DMEM; Solarbio, Beijing, China) supplemented with 15% foetal bovine serum (Sijiqing, Hangzhou, China). HCC cells were seeded onto 96‐well plates at a density of 5 × 10^3^ cells/well or 6‐well plates at 6 × 10^5^ cells/well. After 12 h incubation, the cells were divided into control, SSPH I and SSPH I + inhibitors groups. For the SSPH I groups, cells were treated with 0, 2, 4 or 8 μM SSPH I in the CCK8 assay; 0, 4 or 6 μM for ROS detection and ELISA; and 0 or 4 μM in other assays. For the SSPH I + inhibitors group, cells were pre‐treated with 0.5 μM ferrostatin‐1 (Fer) (Selleck Chemicals), 15 μM U0126 (U0) (Selleck Chemicals, Shanghai, China) or 10 mM N‐acetyl cysteine (NAC) (Selleck Chemicals) for 30 min prior to SSPH I treatment.

### 2.3 CCK8 Assay

HCC cells were processed as mentioned previously and incubated for 24 h. Then, 10 μL of CCK‐8 (Dojindo, Shanghai, China) was added into each well, and the optical density (OD) value was measured at 450 nm following the manufacturer's instructions. All experiments were repeated 3 times.

### 2.4 Flow Cytometry Analysis

HCC cells were processed as mentioned previously and incubated for 24 h. Cells were then stained with PE‐FITC (Solarbio) according to the manufacturer's instructions to detect apoptosis or with a cell cycle analysis kit (Solarbio) according to the manufacturer's instructions to assess cell cycle progression. All experiments were repeated 3 times.

### 2.5 JC‐1 Staining

HepG2 cells were processed as mentioned previously and incubated for 8 h. The mitochondrial membrane potential (MMP) was measured using the JC‐1 staining kit (Thermo Scientific, Shanghai, China), following the manufacturer's instructions. JC‐1 polymers were identified at 550/590 nm (excitation/emission), while JC‐1 monomers were identified at 485/535 nm (excitation/emission). The JC‐1 polymer/monomer ratio was used as an indicator of mitochondrial function.

### 2.6 Transmission Electron Microscope

Cells or tumour tissues were fixed with 2.5% glutaraldehyde for 12 h. After fixation, the cells were postfixed in 1% OsO4 and dehydrated in a series of graded ethanol solutions (25%–100%) and embedded in epoxy resin. Ultrathin sections of the cell samples were stained with 1% uranyl acetate for 15 min and 1% lead citrate for 6 min and observed under TEM (HT‐7800, Hitachi, Japan) at 80 kV.

### 2.7 Western Blot

Total protein of cells or tumour tissue was extracted using RIPA with PMSF and phosphatase inhibitors (Beyotime, Shanghai, China). The concentration of protein was determined using the BCA method. Equal amounts of the protein were loaded and electrophoresed in 10% SDS‐polyacrylamide gels. After being separated by SDS‐PAGE gels, the protein was transferred onto polyvinylidene difluoride membranes. The membranes were blocked with 5% BSA at room temperature for 1 h. Then, the membranes were incubated overnight at 4°C with primary antibodies. The membranes were washed 5 times with TBST and incubated at room temperature with the secondary antibody for 2 h. Protein bands were visualised using enhanced chemiluminescence Western Detection System (Bio‐Rad, Hercules, CA, USA). GAPDH was used as the load control in cell samples, and β‐actin was used as the load control in animal samples.

### 2.8 Immunofluorescence

HepG2 cells were processed as mentioned before and incubated for 8 h. Then cells were washed twice with PBS and fixed with 4% paraformaldehyde for 30 min. Subsequently, cells were washed and incubated with 0.1% Triton X‐100 for 10 min and blocked with 5% BSA for 1 h. Primary antibodies (p‐Erk1/2, Nrf2, HO1) were incubated with the cells at 4°C overnight. Then, the cells were washed three times with PBS and incubated with the secondary antibody (goat anti‐rabbit, Beyotime, Shanghai, China) to visualise the green fluorescence. The nuclei were labelled with DAPI and the images were taken using fluorescence microscopy (Zeiss LSM 710 META, Jena, Germany).

### 2.9 Level of ROS, Fe^2+^, Malondialdehyde (MDA) and Glutathione (GSH)

Intracellular ROS was measured using Reactive Oxygen Species Assay Kit (Beyotime, Shanghai, China). MDA, Fe^2+^ and GSH were measured by ELISA according to the manufacturer's instructions (Beyotime, Shanghai, China). All experiments were repeated three times.

### 2.10 Animals and Tumour Xenograft Models

Male BALB/c nude mice (4–6 weeks, China Shanghai SLAC Laboratory Animal Co., Shanghai, China) were fed in a specific pathogen‐free vivarium under standard conditions. All animal study protocols were approved by the Animal Ethics Committee of the Guangxi University of Chinese Medicine (China). A 50 μL volume of HepG2 cell suspension (1 × 10^7^ cells) was injected into the right flank of each mouse. Tumour volume and body weights were measured every 3 days. Tumour volumes were calculated using the following formula: volume (cm^3^) = 0.5 × L × W^2^. Once the tumour volume reached 50–100 mm^3^, mice were divided into control, SSPH I (30 mg/kg/day, gavage), U0126 (10.5 mg/kg/day, intraperitoneal injection) and SSPH I + U0126 group (*n* = 5). In the SSPH I + U0126 group, U0 was injected 30 min prior to SSPH I. On day 15 after administration, mice were euthanized, and the tumours were weighed and photographed. The synergy between SSPH I and U0126 was calculated using the following formula: Q = Inhibitory_(A+B)_/(Inhibitory_A_ + Inhibitory_B_—Inhibitory_A_ × Inhibitory_B_). Q ≥ 1.15 was considered synergistic, 1.15 > Q > 0.85 was considered additive, and Q ≤ 0.85 was considered antagonism.

### 2.11 Immunohistochemistry

Tumours were fixed in 4% paraformaldehyde overnight and embedded in paraffin. Then tumours were sectioned, de‐paraffinised with xylene and rehydrated. Antigen retrieval was then achieved by incubating the sections with sodium citrate buffer for 15 min. Endogenous peroxidase activity was blocked with 3% hydrogen peroxide under light‐shielded conditions and washed with PBS three times to remove excess hydrogen peroxide. Then the sections were blocked with 5% BSA at room temperature for 1 h, primary antibodies were added and incubated overnight at 4°C. Then, HRP‐labelled secondary antibody (goat anti‐rabbit, Abcam, Shanghai, China) was added for 1 h at room temperature. Finally, DAB chromogen solution was added and counterstained with haematoxylin. The microscopic images were captured using a microscope.

### 2.12 Statistical Analysis

SPSS 25.0 software (SPSS Inc., Chicago, IL) was applied to analyse all experimental data. For multiple groups, ANOVA (with Tukey correction) was performed to compare differences between groups. A value of *p* < 0.05 indicated statistical significance.

## 3 Results

### 3.1 Fer, U0 and NAC Significantly Attenuated SSPH I‐Mediated Anti‐Proliferative Effects in HCC Cells, Concomitantly Modulating Its Pro‐Apoptotic Activity and G2/M Phase Arrest

**FIGURE 1:**
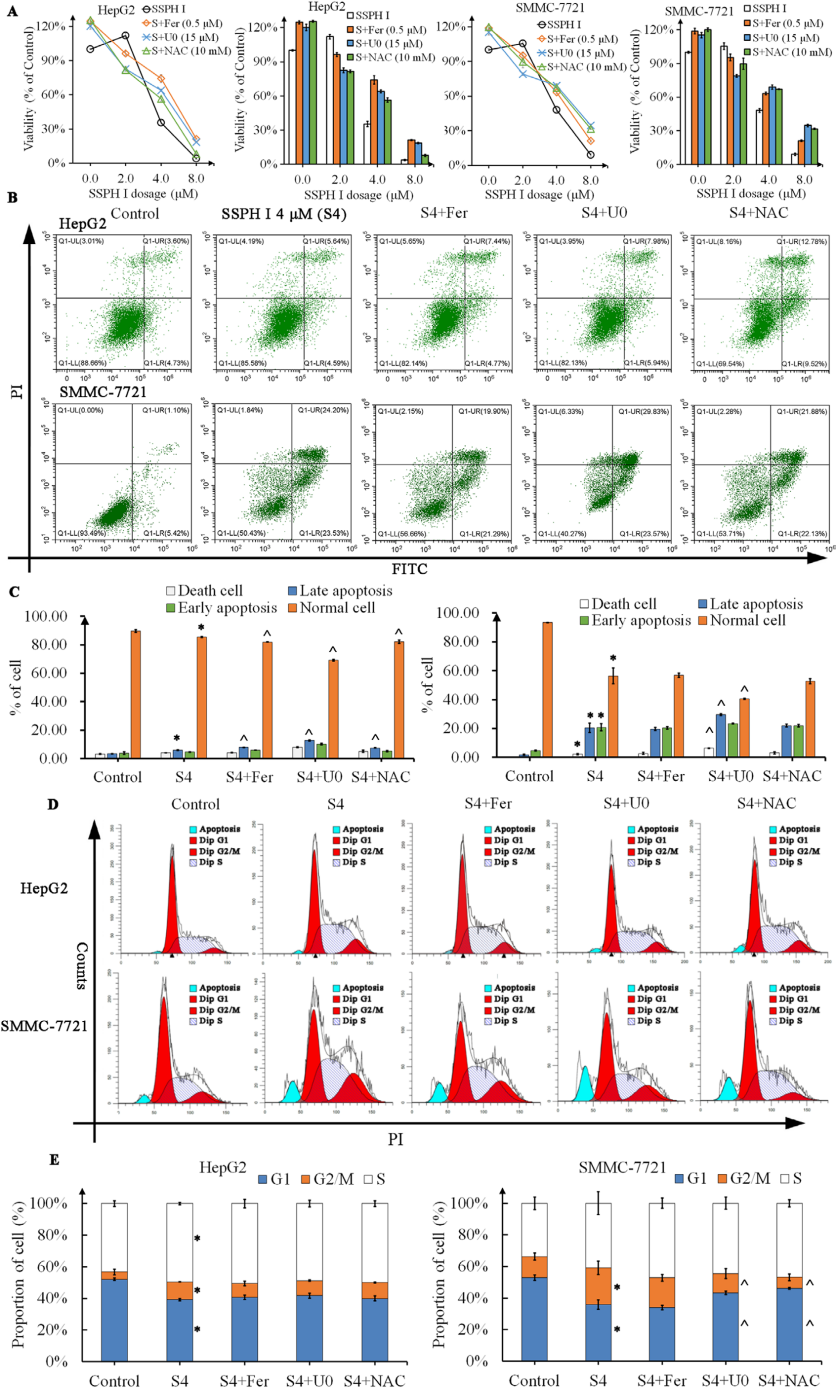
Fer, U0 and NAC interfered with the anti‐proliferation, apoptosis induction and cell cycle interruption effect of SSPH I in HCC cells. (A) SSPH I significantly inhibited the proliferation of HCC cells after 24 h treatment; both Fer, U0 and NAC showed antagonist effects towards SSPH I. (B) Apoptosis was analysed by Annexin V‐FITC/PI staining. (C) SSPH I induced apoptosis in HCC cells after 24 h treatment; Fer, U0 and NAC did not protect HCC cells from apoptosis. (D) Cell cycle analysis was performed by flow cytometry. (E) SSPH I induced cell cycle arrest in HCC cells after 24 h treatment; U0 and NAC rescued SMMC‐7721 cells from G2/M arrest but did not affect HepG2 cells. Data represent the mean ± SD, *n* = 3. *p < 0.05 compared with control; ^*p* < 0.05 compared with SSPH 4 μM.

As shown in Figure 1, SSPH I significantly inhibited the proliferation of HepG2 and SMMC‐7721 cells after 24 h treatment; the IC50 of SSPH I was 3.405 and 3.506 μM for HepG2 and SMMC‐7721 cells. Co‐treated with Fer, U0 and NAC significantly reduced the anti‐proliferation effect of SSPH I on HCC cells, indicating that ferroptosis, phosphorylation of Erk1/2 and ROS are involved in the inhibition of SSPH I on HCC cells.

We further detected the apoptosis‐induction effect of SSPH I on HCC cells. At 4 μM, SSPH I induced minimal apoptosis in HepG2 cells, suggesting non‐apoptotic dominance in its anti‐HepG2 activity (Figure 1B,C). Different from HepG2, SSPH I 4 μM induced significantly early and late apoptosis in SMMC‐7721 cells (*p* < 0.05). Interestingly, none of the Fer, U0 or NAC decreased the apoptotic induction of SSPH I, but rather increased the apoptotic ratio. Co‐treatment with Fer, U0 or NAC elevated late apoptotic populations while decreasing viable cell fractions in HepG2 cells (*p* < 0.05). In SMMC‐7721 cells, only U0 significantly increased the proportion of apoptotic and dead cells (*p* < 0.05). These results indicated Fer, U0 and NAC do not protect HCC cells by inhibiting apoptosis, but contribute to the crosstalk of apoptosis and other types of cell death.

SSPH I significantly blocked HepG2 and SMMC‐7721 cells in G2/M phase (*p* < 0.05), and the proportion of HepG2 cells in the S phase also increased (*p* < 0.05). Co‐treatment with U0 and NAC rescued the SMMC‐7721 cells from G2/M arrest (*p* < 0.05). However, none of the Fer, U0 or NAC restored the cell cycle blocked by SSPH I in HepG2 cells.

Taken together, these results indicated that ferric ion, Erk1/2 and ROS are elements of the inhibitory effect of SSPH I on HCC cells and partially participate in the regulation of SSPH I‐induced apoptosis and cell cycle arrest.

### 3.2 Fer, U0 and NAC Alleviated the Mitochondrial Damage Induced by SSPH I

To elucidate the function of Erk1/2, Fe^2+^ and ROS in mitochondrial damage induced by SSPH I, MMP and mitochondrial morphology were evaluated. JC‐1 fluorescent probe was used to detect the MMP. Fluorescence of JC‐1 monomer (green) was significantly increased after SSPH I 4 μM 8 h treatment, with diminished JC‐1 polymer (red) fluorescence intensity. Both Fer, U0 and NAC rescue the SSPH I‐induced decrease in MMP.

We further observed the mitochondrial ultrastructure using a transmission electron microscope. Increased density of the membrane, cristae fragmentation with reduced mitochondrial length were observed in HepG2 cell's mitochondria after SSPH I 4 μM 8 h treatment; part of the mitochondria showed dissolution of the double membrane. Neither Fer, U0, nor NAC obviously affect the length and cristae of mitochondria; however, increased density of the membrane was observed after U0 treatment. Co‐treatment with Fer, U0 or NAC increased the length of mitochondria but failed to show significance compared with SSPH I alone. An increase in mitochondrial cristae was observed in Fer, U0 and NAC co‐treated groups; meanwhile, a decrease in membrane density was observed only in the Fer co‐treated group.

**FIGURE 2:**
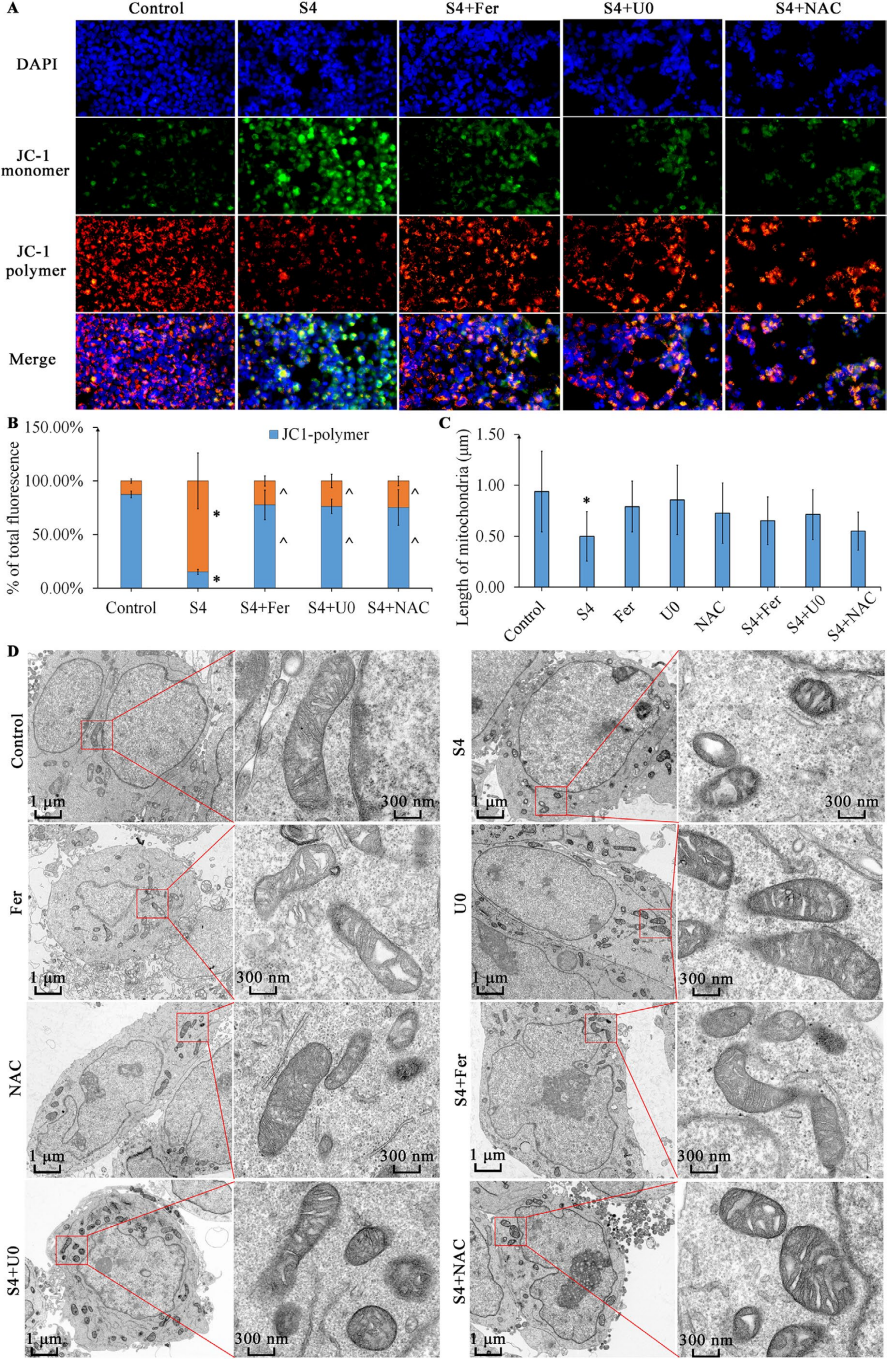
Fer, U0 and NAC alleviated the mitochondrial damage induced by SSPH I in HepG2 cells. (A) JC‐1 fluorescent probe detects MMP in HepG2 cells after 8 h treatment. (B) Ratio of fluorescence of JC‐1 monomer and polymer. (C) Length of mitochondria in HepG2 cells after 8 h treatment. (D) TEM observations of mitochondrial ultrastructure in HepG2 cells. Data represent the mean ± SD, B *n* = 3; C *n* = 20. *p < 0.05 compared with control; ^*p* < 0.05 compared with SSPH 4 μM.

Collectively, the above findings suggest that blocking Erk1/2, decreasing ferric ion or ROS can alleviate the mitochondrial damage induced by SSPH I; the variance in mitochondrial morphology indicates distinct mechanisms (Figure 2).

### 3.3 Inhibition of Erk1/2 Phosphorylation Totally Blocked the Effect of SSPH I on Nrf1/2‐HO1 and Ferroptosis‐Related Proteins

**FIGURE 3:**
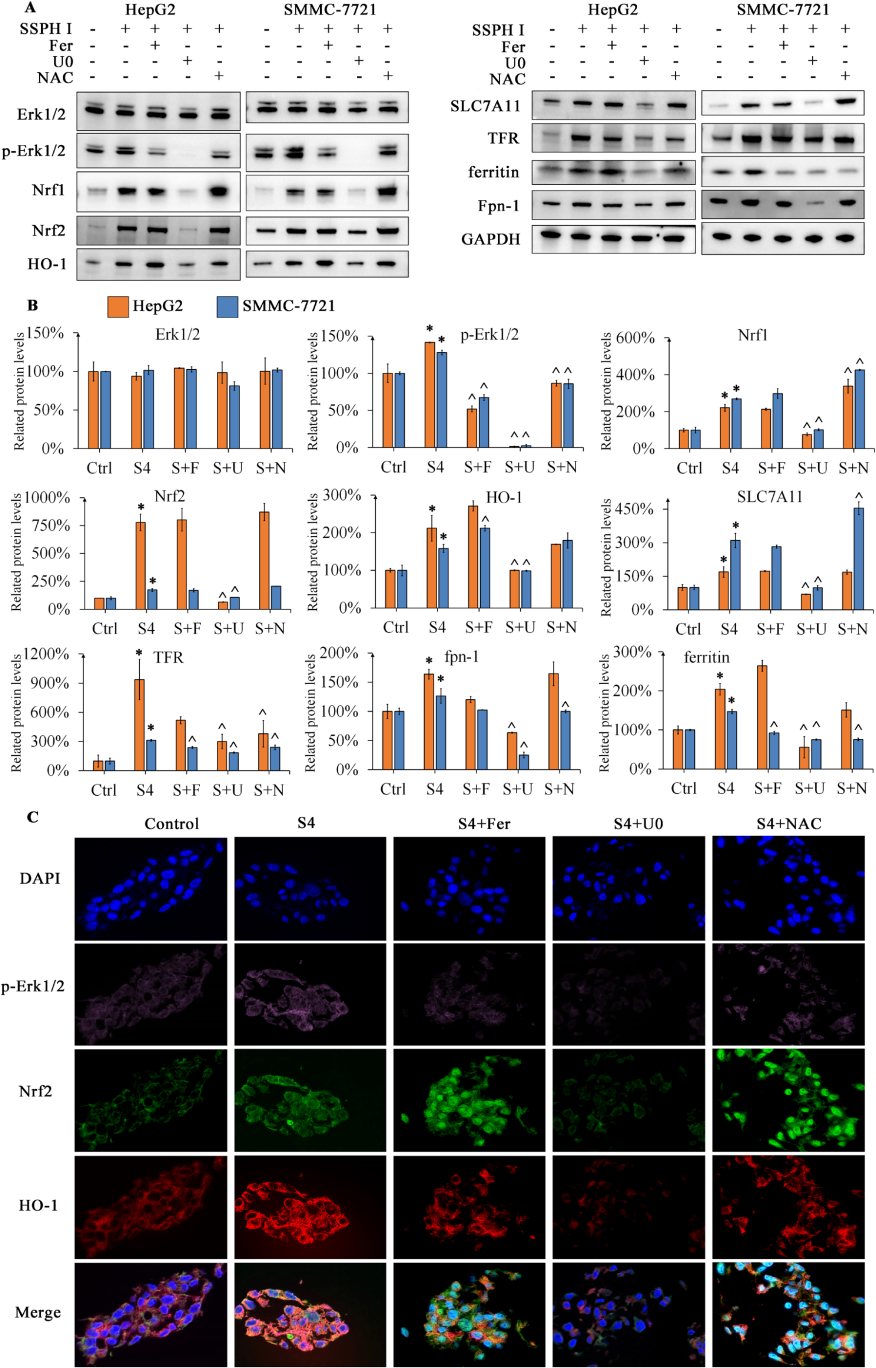
Fer, U0 and NAC regulate protein expression induced by SSPH I. (A) The protein levels of HCC cells after 8 h treatment were measured. (B) SSPH I upregulated Nrf1/2‐HO‐1 and ferroptosis‐relative proteins; the combination of Fer, U0 and NAC further regulated the protein expression. (C) Immunofluorescence verified the expression of p‐Erk1/2, Nrf2 and HO‐1 in HepG2 cells. Cell shrinkage was observed after SSPH I treatment. The combination of Fer, U0 and NAC did not relieve the cell shrinkage. The increased fluorescence in the nucleus indicated that Fer and NAC facilitate the translocation of Nrf2. Data represent the mean ± SD, *n* = 2. *p < 0.05 compared with control; ^*p* < 0.05 compared with SSPH 4 μM.

To investigate the association between Erk1/2, ferroptosis and mitochondrial damage, we measured the expression of ferroptosis‐related proteins and Nrf1/2‐HO1. Erk1/2‐Nrf1/2‐HO‐1 axis activation typically exerts a protective effect on mitochondrial function [8, 20]. SSPH I significantly induced Erk1/2 phosphorylation (*p* < 0.05) without upregulating Erk1/2 in HCC cells. Concurrently, Nrf1/2 and HO‐1 were also upregulated (*p* < 0.05), suggesting mitochondrial damage in these cells. Both Fer and NAC normalised Erk1/2 phosphorylation induced by SSPH I, demonstrating that ferric ion and ROS are involved in the phosphorylation of Erk1/2. However, the activation of Nrf1/2 and HO‐1 was not cancelled after Erk1/2 normalisation. Instead, Nrf1 was further upregulated after co‐treatment with NAC (*p* < 0.05), demonstrating that Nrf1/2‐HO1 is not directly regulated by Erk1/2. In contrast to Fer and NAC, the phosphorylation of Erk1/2 was blocked completely by U0, thus totally normalising the expression of Nrf1/2 and HO‐1 induced by SSPH I. As shown in Figure 3C, Fer, U0 and NAC showed similar results to WB. Additionally, we can find that Fer and NAC facilitated the translocation of Nrf2 into the nucleus. Previous studies have shown that chemical inhibition of the Erk1/2‐Nrf2 axis aggravates oxidative stress and mitochondrial damage [8]. Interestingly, blocking Erk1/2‐Nrf1/2‐HO‐1 induced by SSPH I markedly enhanced MMP and viability, demonstrating that Erk1/2 determines the downstream effect of SSPH I.

We next analysed ferroptosis‐related proteins. In HCC cells, TFR was significantly upregulated following SSPH I treatment. To counteract the upregulation of TFR caused by SSPH I, ferritin, fpn‐1, and SLC7A11 were upregulated to store and expel ferric ions while promoting cystine uptake. Co‐treatment with Fer downregulated TFR and ferritin in SMMC‐7721 cells (*p* < 0.05) and had no significant effect on these proteins in HepG2 cells. Fer did not modulate SSPH I‐induced SLC7A11 or FPN1 expression. On the other side, NAC downregulated SSPH I‐induced TFR in both HepG2 and SMMC‐7721 cells but had no effect on SLC7A11, fpn‐1 or ferritin levels in HepG2 cells. SLC7A11 was significantly increased by NAC in SMMC‐7721 cells, accompanied by the downregulation of fpn1 and ferritin (*p* < 0.05). Previous studies have extensively demonstrated the anti‐ferroptosis effect of Fer and NAC [21, 22]; however, the relationship between Erk1/2 and ferroptosis is rarely mentioned. A study suggested that Erk1/2 activation can maintain the expression of ferritin [14]. In our study, co‐treatment with U0 downregulated or abolished the effects of SSPH I on ferroptosis‐related proteins, highlighting the dominant role of Erk1/2 in SSPH I‐induced ferroptosis.

From the results, we can speculate that ferric ions and ROS participated in the activation of Erk1/2 but are not key factors in the activation of Nrf1/2 and HO‐1. While NAC and Fer counteract SSPH I‐induced ferroptosis and oxidative stress through distinct mechanisms, Erk1/2 ultimately serves as the upstream regulator of SSPH I's downstream effects.

### 3.4 Fer, U0 and NAC Assisted Cells Against SSPH I‐Induced Oxidative Stress and Ferroptosis

To further analyse the iron overload and oxidative stress in HCC cells, intracellular ROS, MDA, Fe^2+^ and GSH were measured after an 8 h treatment. SSPH I caused significant ROS, MDA and Fe^2+^ accumulation (*p* < 0.05) while depleting GSH levels in HCC cells (*p* < 0.05). Both Fer and U0 exhibited antagonistic effects against SSPH I (*p* < 0.05). NAC significantly reduced SSPH I‐induced ROS generation and MDA accumulation in HCC cells (*p* < 0.05) but did not affect Fe^2+^ accumulation. NAC significantly increased GSH in SMMC‐7721 cells (*p* < 0.05), but not in HepG2 cells. This discrepancy may be attributed to NAC‐induced upregulation of SLC7A11 expression.

**FIGURE 4:**
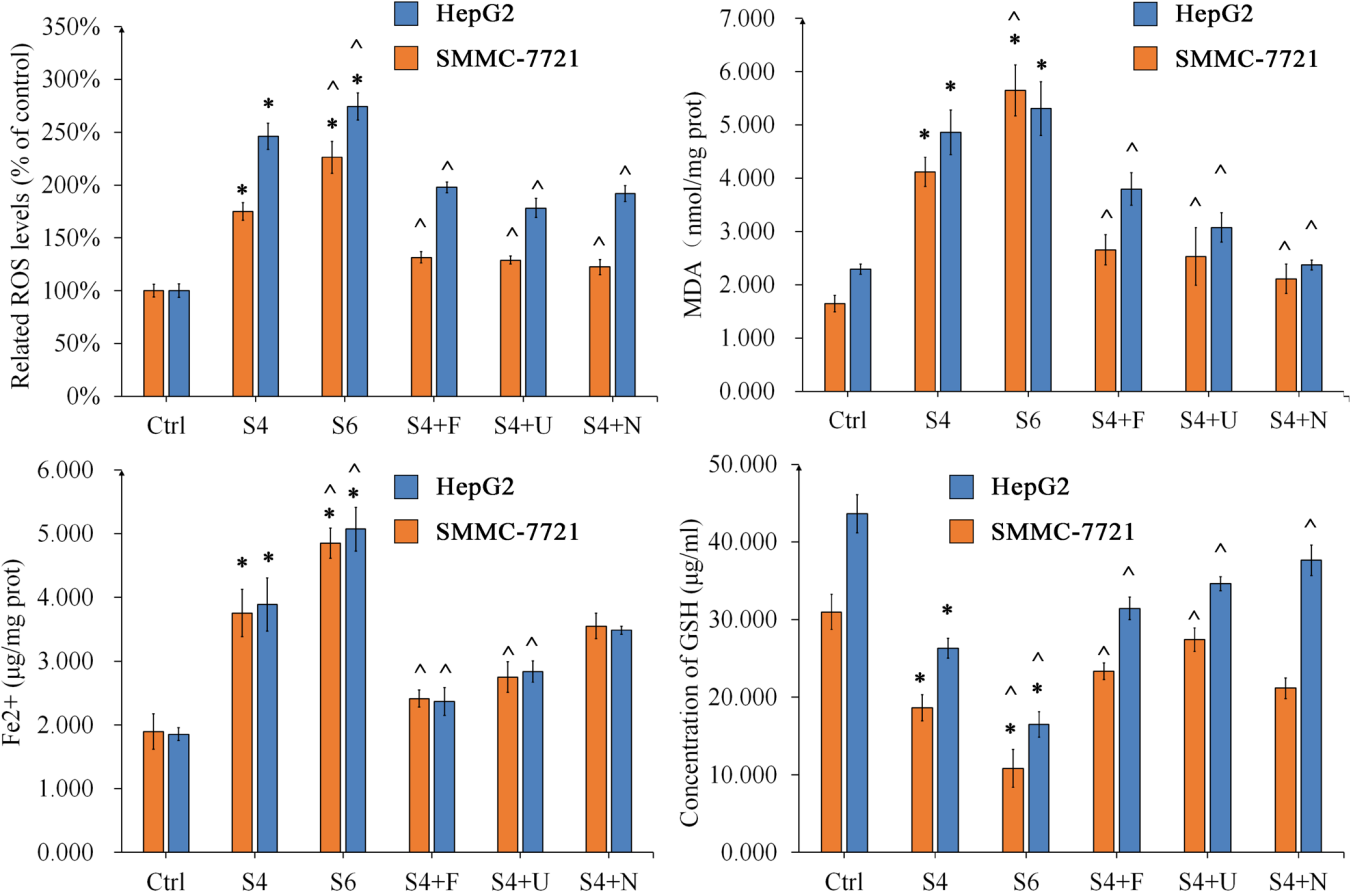
The protective role of Fer, U0 and NAC in SSPH I induced oxidative stress and ferroptosis in HCC cells. Data represent the mean ± SD, *n* = 3. *p < 0.05 compared with control; ^*p* < 0.05 compared with SSPH 4 μM.

These results suggest distinct mechanisms of action: Fer primarily counteracts oxidative stress and ferroptosis through iron chelation and modulation of iron transport proteins; NAC exerts its effects via ROS scavenging and enhancement of SLC7A11 activity; whereas U0 uniquely inhibits Fe^2+^ and ROS production by targeting specific regulatory proteins (Figure 4).

### 3.5 Both SSPH I and U0 Inhibited Xenograft Tumour Growth In Vivo, but Exhibited Antagonistic Effects When Administered in Combination

**FIGURE 5:**
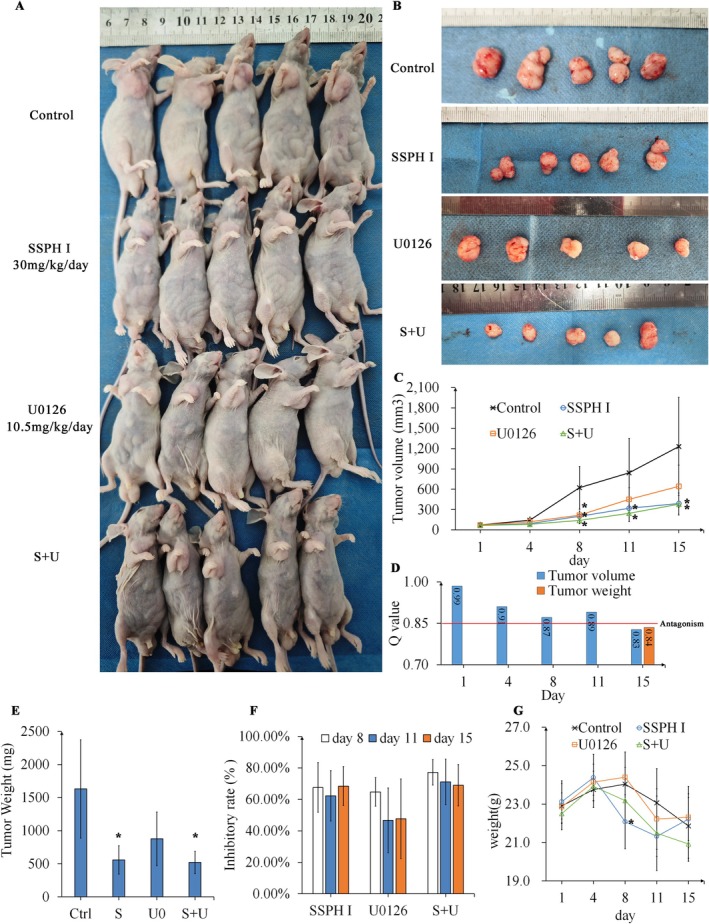
SSPH I and U0 inhibited the growth of HepG2 xenograft tumours. (A) Representative photographs of xenograft tumours from each dosage group in HepG2 nude mice models. (B) Gross morphology of excised HepG2 xenograft tumours. (C) Tumour growth curves of HepG2 xenografts. (D) *Q* value of volume and weight of tumours. (E) Final tumour weights of HepG2 xenografts. (F) Tumour growth inhibition rates across treatment groups. (G) Body weight changes of nude mice during treatment. Data represented the mean ± SD, *n* = 5. *p < 0.05 compared with control.

To investigate the therapeutic efficacy and mechanism of SSPH I in vivo, HepG2 cells were subcutaneously implanted into the right flank of nude mice to establish xenograft tumour models. SSPH I treatment significantly inhibited xenograft tumour growth (*p* < 0.05), with no significant differences observed in body weights between groups. Following 15 days of SSPH I treatment, both tumour volume and weight showed significant reduction compared to the control group (*p* < 0.05). U0 monotherapy demonstrated significant tumour growth inhibition on day 8 (*p* < 0.05). However, tumour volume rebounded rapidly by days 11 and 15, ultimately showing no statistically significant differences in final tumour volume (*p* = 0.149) or weight (*p* = 0.071) compared to the control group. The SSPH I + U0 combination group showed comparable tumour volume and weight inhibition to SSPH I monotherapy. Notably, while the drug combination exhibited additive effects on days 8 and 11, this interaction transitioned to antagonism by day 15, with no overall synergistic effect observed throughout the treatment period. Previous investigations demonstrate that U0 inhibits HepG2 xenograft. Notably, the decreased tumour suppression rate of U0 following the progression of xenograft volumes could also be observed in this study [23]. Furthermore, analyses of other Erk1/2 inhibitors reveal enhanced anti‐tumour efficacy when treatment initiates before tumour formation, contrasting with reduced inhibitory effects on established hepatocellular carcinoma xenografts [24]. These findings provide indirect mechanistic support for the antagonistic outcomes observed following 15‐day combination therapy with U0126 and SSPH I. Collectively, our study provides the first experimental evidence confirming SSPH I's anti‐HCC efficacy in vivo, while revealing U0's antagonistic interaction with SSPH I in this setting (Figure 5).

### 3.6 SSPH I and U0 Caused Morphological Changes and Regulated Nrf1/2‐HO‐1 and Ferroptosis‐Related Proteins in HepG2 Xenograft Tumours

**FIGURE 6:**
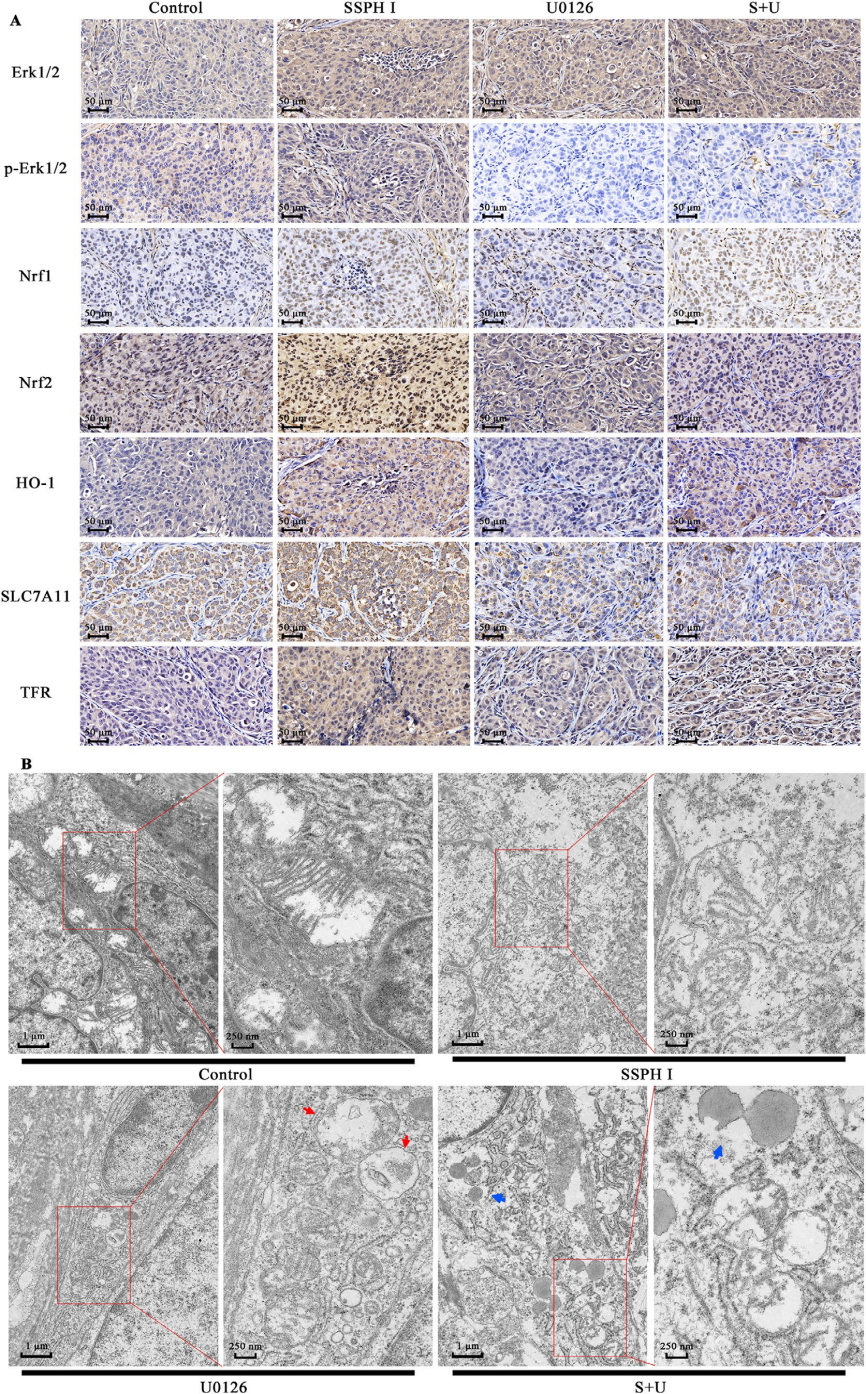
SSPH I and U0 modulate protein expression levels and mitochondrial morphology in HepG2 xenograft tumours. (A) Protein expression levels in tumour tissues were visualised by immunohistochemical staining. (B) Mitochondrial morphology in tumour tissues was examined by transmission electron microscopy. Red arrows: Autophagic vacuoles; blue arrows: Autophagic vacuoles containing lipid droplets.

As shown in Figure 6, tumour cells in the control group exhibited tightly packed arrangements with well‐defined intercellular borders. SSPH I‐treated tumours displayed cytoplasmic vacuolization, blurred cellular edges and extensive necrotic foci, accompanied by tissue shrinkage. In contrast, the U0 monotherapy group showed less extensive necrosis compared to SSPH I treatment. Instead, U0‐treated tumours presented as fragmented tissue masses characterised by cellular rupture, cytoplasmic leakage and vacuolisation. Notably, the SSPH I + U0 combination group exhibited morphological features more closely resembling those of the U0 monotherapy group. The combination group demonstrated fragmented tumour masses with cellular rupture and cytoplasmic vacuolization but displayed reduced necrotic foci compared to SSPH I monotherapy.

Consistent with in vitro findings, SSPH I treatment upregulated the expression of p‐Erk1/2, Nrf1/2, HO‐1, SLC7A11 and TFR in vivo. SSPH I promoted nuclear translocation of Nrf1 and increased Nrf2 levels in both cytoplasmic and nuclear compartments. U0 suppressed Erk1/2 phosphorylation but did not significantly alter the expression of other investigated proteins. U0126 attenuated SSPH I‐induced upregulation of p‐Erk1/2, Nrf2 and SLC7A11, and additionally reduced nuclear Nrf2 accumulation. These protein expression patterns were subsequently confirmed by western blot analysis.

In the control group, tumour tissue exhibited mitochondria with tightly packed, well‐organised cristae. Rounded mitochondria, and lesser and unarranged mitochondrial cristae were observed after SSPH I treatment, accompanied by extensive disruption of cellular ultrastructure. The U0 monotherapy group similarly showed rounded mitochondria with reduced cristae density, but distinctively displayed abundant autophagic vacuoles within tumour cells. Notably, the SSPH I + U0 combination failed to ameliorate SSPH I‐induced mitochondrial damage. Instead, it exacerbated ultrastructural destruction, manifesting as complete disintegration of mitochondrial inner membranes and autophagic vacuoles containing lipid droplets. Whether lipophagy contributes to the observed antagonism between SSPH I and U0 requires further investigation. Prior research has established that SSPH I suppresses autophagy in HCC cells through inhibition of autophagosome‐lysosome fusion, whereas U0 counteracts this effect by attenuating SSPH I‐induced accumulation of LC3‐I, LC3‐II and p62 [17]. These data suggest that although U0 mechanistically antagonises SSPH I, chronic SSPH I administration may perpetuate mitochondrial injury. U0 appears to mitigate further tissue damage by redirecting cellular fate towards apoptosis and autophagic pathways.

### 3.7 Erk1/2 Phosphorylation Played a Crucial Role in SSPH I‐Induced Oxidative Stress and Ferroptosis In Vivo

**FIGURE 7:**
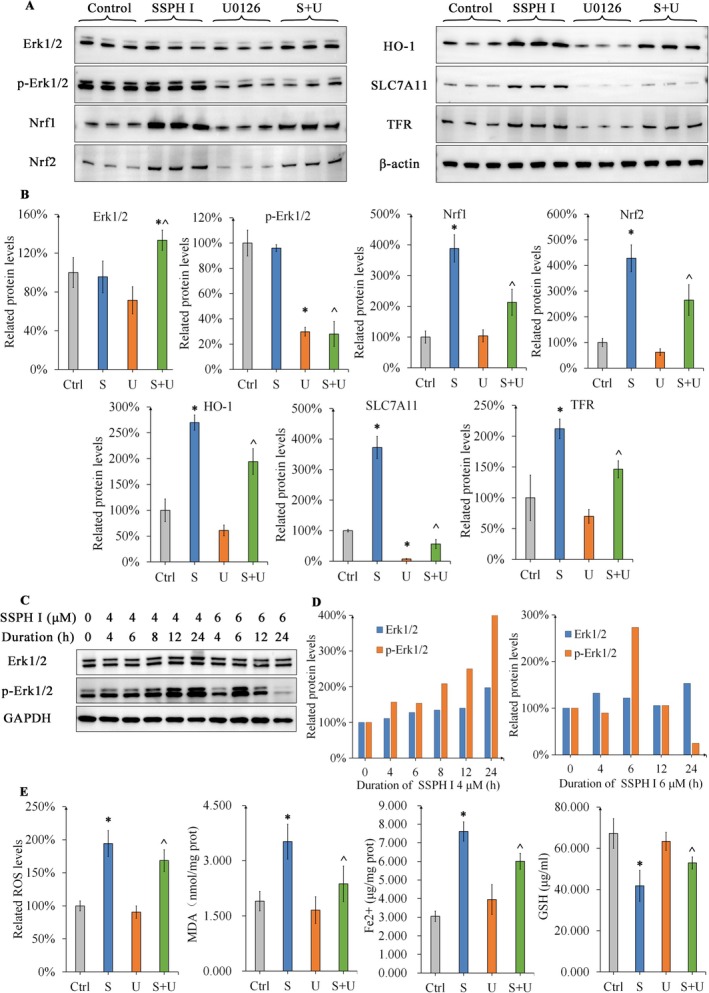
SSPH I and U0 modulate protein expression and the concentrations of ROS, MDA, GSH and Fe^2+^ in HepG2 xenograft tumours. (A) Protein expression in tumour tissues was analysed by Western blotting. (B) SSPH I activated Erk1/2 and upregulated Nrf1/2, HO‐1, SLC7A11 and TFR; U0 antagonised the effects of SSPH I by inhibiting Erk1/2 phosphorylation. (C and D) The duration and concentration of SSPH I influence Erk1/2 phosphorylation. (E) U0 attenuates SSPH I‐induced oxidative stress and ferroptosis in vivo. (B and E), data represent the mean ± SD, *n* = 3. *p < 0.05 compared with control; ^*p* < 0.05 compared with SSPH I group.

Protein expression, Fe^2+^ levels and oxidative stress levels were measured to clarify the interaction between SSPH I and U0. Similar to the in vitro experiments, the expression of Nrf1/2, HO‐1, SLC7A11 and TFR in HepG2 xenograft tumours was significantly increased after SSPH I treatment. However, no significant changes in p‐Erk1/2 levels were observed. In a previous study, we observed that Erk1/2 phosphorylation varied depending on SSPH I concentration or treatment duration [25]. As expected, 4 μM SSPH I continuously activated Erk1/2 over a 24‐h period. Furthermore, Erk1/2 activation peaked after 6 h of treatment with 6 μM SSPH I, followed by a decline and subsequent inhibition (Figure 7C,D). These results indicated that the undefined pharmacokinetic properties of SSPH I in vivo may be related to unaltered p‐Erk1/2 levels. On the other side, U0 partially inhibited Erk1/2 phosphorylation in vivo, leading to a significant decrease in SLC7A11 expression (*p* < 0.05). Similar to the in vitro experiment, U0‐mediated blockade of Erk1/2 phosphorylation normalised the SSPH I‐induced expression of Nrf1/2, HO‐1, SLC7A11, and TFR, demonstrating that Erk1/2 is a key pathway in SSPH I‐mediated protein regulation in vivo.

To further investigate SSPH I‐induced oxidative stress and ferroptosis in vivo, the levels of ROS, MDA, GSH, and Fe^2+^ in tumour tissues were measured. SSPH I significantly increased ROS, MDA and Fe^2+^ levels (*p* < 0.05), resulting in GSH depletion in tumour tissues (*p* < 0.05). Compared to the SSPH I group, U0 did not regulate tissue ROS, MDA, GSH or Fe^2+^ levels as reported previously [26], but exhibited antagonistic effects against SSPH I (*p* < 0.05). Collectively, these findings suggest that SSPH I and U0 exerted comparable effects in vivo and in vitro. Mechanistically, Erk1/2 blockade reduced SSPH I‐induced oxidative stress and ferroptosis in vivo.

## 4 Discussion

Erk1/2 is significantly upregulated in HCC compared to normal liver tissues, serving dual roles in sustaining mitochondrial homeostasis and counteracting oxidative stress [3, 27]. Driven by inherent mitochondrial dysfunction and chronic oxidative burden, HCC cells exhibit heightened reliance on glycolysis to adapt to their hypoxic microenvironment [28, 29], coupled with suppressed Nrf2 expression [30]. By regulating Erk1/2 or Nrf2, various natural products can induce oxidative damage or ferroptosis in HCC cells [11, 15]. In stark contrast, normal hepatocytes leverage Erk1/2–Nrf2 activation as a cytoprotective mechanism against oxidative injury [8], highlighting a therapeutically exploitable dichotomy between physiological repair and oncogenic adaptation. However, whether Erk1/2 acts as the initiator of oxidative stress in HCC pathogenesis, and whether its activation exerts pro‐ferroptotic or anti‐ferroptotic effects in HCC remain unclear. These mechanisms need to be clarified before the development of Erk1/2 targeting anti‐HCC drugs.

SSPH I ((25S)‐spirost‐5‐en‐3β‐yl‐O‐α‐ʟ‐rhamnopyranosyl‐(1 → 2)‐O‐[O‐β‐D‐glucopyranosyl‐(1 → 4)‐α‐ʟ‐rhamnopyranosyl‐(1 → 3)]‐β‐D‐glucopyranoside; CAS: 290809‐72‐2), a steroidal saponin with established anticancer properties, was herein demonstrated for the first time to exert potent anti‐HCC effects in vivo. In a previous study, we found that the anti‐proliferation, oxidative stress induction effect of SSPH I is co‐related to Erk1/2 phosphorylation [17, 31]. In addition, SSPH I provokes iron accumulation and ferroptosis in HepG2 cells via a novel mechanism characterised by TFR upregulation in the absence of SLC7A11/SLC7A5 inhibition [16]. Likewise, our experiment showed that SSPH I activated Erk1/2, oxidative stress and ferroptosis in HCC cells and further disclosed the relationship between Erk1/2 and ferroptosis in vivo and in vitro. The effect of SSPH I gave us a gateway to analyse the connections of Erk1/2 activation, oxidative stress and ferroptosis.

The functional interplay between Erk1/2 and ferroptosis in both hepatic malignancies and physiological contexts remains poorly characterised, with conflicting reports in the literature. A study implies that activation of Erk1/2 assists HepG2 cells against ferroptosis [32]. Another study indicated that Erk1/2 promotes ferritinophagy in HCC under Nrf2 inhibition [14]. Emerging evidence implicates Erk1/2 in ferroptosis regulation across diverse pathologies, including NSCLC, cardiovascular diseases and neurodegenerative disorders. In these studies, Erk1/2 may regulate SLC7A11, FTH1, FTL and SOD expression. However, the role of Erk1/2 in ferroptosis still remains inconsistent [13, 14, 33, 34]. Our study found that the expression of SLC7A11 in HCC is highly dependent on the activation of Erk1/2. Generally, inhibition of SLC7A11 will exacerbate ferroptosis within the HCC cells [35]. Since SSPH I did not inhibit SLC7A11, Erk1/2 inhibition failed to exacerbate GSH depletion in HCC cells. Secondly, TFR is the key protein of SSPH I‐induced ferroptosis. Fer and NAC partially antagonised S‐induced ferroptosis, but only Erk1/2 normalised the SSPH I‐induced ferroptosis, thus normalising the fpn1, ferritin and Fe^2+^. In general, System $X_{c}^{-\cdot }$ targeting ferroptosis inducers exhibit therapeutic synergy with Erk1/2 inhibitors, whereas iron chelators counteract Erk1/2 blockade efficacy, underscoring the necessity for mechanistic stratification in combination therapy design.

The Erk1/2‐Nrf2‐HO1 axis is a conserved cytoprotective mechanism against oxidative stress and mitochondrial dysfunction across multiple tissue types [8, 27, 36]. Recently, a study revealed that the balance of Nrf1 and Nrf2 is an indispensable redox‐determining factor for mitochondrial homeostasis in HCC [20]. In previous studies, NAC treatment may induce a varied range of Nrf1/2‐HO1 expression in liver cells, indicating that the activation of Nrf1/2‐HO1 is more likely induced by oxidative stress and mitochondrial damage than NAC [37, 38]. In our experiment, NAC restored mitochondrial MMP, rescued HCC cells from oxidative stress by downregulating ROS; however, the expression of Nrf2‐HO1 was not downregulated by NAC. In addition, Nrf1 was further elevated following NAC treatment. Nrf2‐HO1 activation also plays a protective role in ferroptosis in cardiovascular and neural tissues [39, 40]. However, Nrf2‐HO1 regulates independently of ferroptosis. The experiment in human renal epithelial HK2 cells showed that Fer did not affect the expression of Nrf2 [41]. Likewise, Fer did not affect Nrf2‐HO1 activated by SSPH I in HCC. How Nrf1/2 affects ferroptosis in HCC is still unclear. Our results revealed no significant correlation between Nrf1/2 expression levels and dynamic fluctuations of ROS or Fe^2+^; it is necessary to block Nrf1/2 to further verify the relationship between Nrf1/2 and ferroptosis in HCC. The regulation of Erk1/2 on Nrf1/2 in HCC has not been discussed. Our experimental evidence has unravelled that Nrf1/2‐HO1 is partially regulated by Erk1/2 in HCC. Blocking Erk1/2 normalised the SSPH I induced Nrf1/2‐HO1 activation. Conversely, U0 alone did not affect the expression of Nrf2‐HO1 [42].

In HCC, it is commonly seen that Erk1/2 and ROS are co‐activated after treatment [5, 43]. Meanwhile, some studies still maintain that ROS accumulation can be induced by inhibiting Erk1/2 [11, 44]. Our study found that SSPH I‐induced Erk1/2 phosphorylation exhibits dose‐ and time‐dependent dynamics, demonstrating that the phosphorylation of Erk1/2 should be verified at different time points and concentrations to avoid incorrect judgement of Erk1/2 activation. In addition, some studies suggested that ROS accumulation leads to the activation of Erk1/2 [6]; similarly, removing ROS by Fer or NAC downregulated p‐Erk1/2 induced by SSPH I. To our surprise, the activation of Nrf1/2‐HO1 and ferroptosis‐related proteins was not normalised under the inhibition of Erk1/2; instead, chemically blocking Erk1/2 before SSPH I exerted a complete blocking effect. The above‐described evidence revealed that Erk1/2 is the start‐point of SSPH I‐induced oxidative stress, mitochondrial damage and ferroptosis.

Apoptotic cell death, autophagy and cell cycle arrest are frequently involved in oxidative cell death [10, 45]. SSPH I induced autophagosome formation but inhibited autophagy flux in HepG2 cells, inhibiting ERK1/2 phosphorylation or combining NAC can reverse autophagy by down regulating LC3‐II [17]. In our results, inhibition of Erk1/2 with U0 caused autophagic vacuoles to appear in xenograft tissues. Paradoxically, augmented autophagic flux failed to potentiate SSPH I's anticancer efficacy, potentially attributable to its dual role in mitochondrial quality control and clearance of ferroptotic lipid peroxides. Since multiple signals such as STAT3 and mTOR are participating in the regulation of autophagy, the mechanism of Erk1/2 in SSPH I regulated autophagy requires further analysis [46]. In previous studies, iron deprivation reprogrammed cell death modalities by enabling crosstalk between SSPH I‐induced ferroptosis and apoptosis in HepG2 cells [16], a phenomenon recapitulated in our experimental system. In addition, inhibition of p‐Erk1/2 usually intensifies apoptosis in HCC cells [47]. Our research found that the combination of U0 resulted in the most significant increase in apoptosis in HCC cells. Furthermore, U0 and NAC rescue the SMMC‐7721 cells from G2/M arrest, which may partially contribute to their protective effect towards HCC cells. Taken together, blocking Erk1/2 shifts SSPH I induced ferroptosis to apoptosis and autophagy, which reduces the inhibitory effect of SSPH I on HCC cells.

This study has several limitations and unresolved issues. Firstly, the effects of SSPH I on mitochondrial dynamics, respiratory function, biogenesis, and autophagy in hepatocellular carcinoma cells require more comprehensive investigation. Secondly, the regulatory significance of SSPH I in mitochondrial function through Erk1/2‐mediated modulation of the Nrf1/2‐HO1 signalling pathway remains to be elucidated. These questions will be systematically addressed in our subsequent research endeavours.

## 5 Conclusions

In conclusion, our findings establish Erk1/2 activation as the central driver of SSPH I‐induced triple cascades—oxidative stress, mitochondrial dysfunction and ferroptosis—in HCC. While ROS scavengers and iron chelators attenuated SSPH I‐induced Erk1/2 phosphorylation and mitigated its downstream effects, they failed to fully abrogate the compound's dual regulation of ferroptosis and oxidative stress. Collectively, our findings position Erk1/2 as a nodal regulator orchestrating the tripartite interplay between ferroptosis susceptibility, Nrf1/2‐mediated redox adaptation and mitochondrial plasticity in HCC.

## Author Contributions

**Yuewen Sun:** conceptualization (lead), data curation (equal), funding acquisition (lead), project administration (equal), supervision (equal), writing – original draft (equal), writing – review and editing (equal). **Ying Zhou:** data curation (equal), formal analysis (equal), investigation (equal), methodology (equal), visualization (equal), writing – original draft (lead). **Dan Huang:** data curation (equal), methodology (equal), writing – review and editing (equal). **Zhiguang Zhao:** resources (equal), software (equal), validation (equal). **Qingrui Shao:** formal analysis (equal), writing – review and editing (equal). **Jianzhe Li:** methodology (equal), writing – review and editing (equal). **Xiaofang Zhao:** formal analysis (equal), resources (equal). **Xudong Liu:** funding acquisition (supporting), project administration (equal), resources (equal), supervision (equal), visualization (equal), writing – review and editing (equal).

## Ethics Statement

All animal study protocols were approved by the Animal Ethics Committee of Guangxi University of Chinese Medicine (China).

## Conflicts of Interest

The authors declare no conflicts of interest.

## Supporting information


Data S1.

## Data Availability

The data used and/or analyzed in the study are available in the Supporting Information (Data S1).

## References

[1] J. D. Yang , P. Hainaut , G. J. Gores , A. Amadou , A. Plymoth , and L. R. Roberts , “A Global View of Hepatocellular Carcinoma: Trends, Risk, Prevention and Management,” Nature Reviews. Gastroenterology & Hepatology 16, no. 10 (2019): 589–604, 10.1038/s41575-019-0186-y. 31439937 PMC6813818

[2] H. Moon and S. W. Ro , “MAPK/ERK Signaling Pathway in Hepatocellular Carcinoma,” Cancers 13, no. 12 (2021): 3026, 10.3390/cancers13123026. 34204242 PMC8234271

[3] L. Lei , G.‐D. Zhao , Z. Shi , L.‐L. Qi , L.‐Y. Zhou , and Z.‐X. Fu , “The Ras/Raf/MEK/ERK Signaling Pathway and Its Role in the Occurrence and Development of HCC,” Oncology Letters 12, no. 5 (2016): 3045–3050, 10.3892/ol.2016.5110. 27899961 PMC5103898

[4] L. Chen , Y. Shi , C. Y. Jiang , L. X. Wei , Y. L. Wang , and G. H. Dai , “Expression and Prognostic Role of Pan‐Ras, Raf‐1, pMEK1 and pERK1/2 in Patients With Hepatocellular Carcinoma,” European Journal of Surgical Oncology 37, no. 6 (2011): 513–520, 10.1016/j.ejso.2011.01.023. 21324414

[5] L. Han , C. Zhang , D. Wang , et al., “Retrograde Regulation of Mitochondrial Fission and Epithelial to Mesenchymal Transition in Hepatocellular Carcinoma by GCN5L1,” Oncogene 42, no. 13 (2023): 1024–1037, 10.1038/s41388-023-02621-w. 36759571

[6] L. Wan , Y. Wang , Z. Zhang , et al., “Elevated TEFM Expression Promotes Growth and Metastasis Through Activation of ROS/ERK Signaling in Hepatocellular Carcinoma,” Cell Death & Disease 12, no. 4 (2021): 325, 10.1038/s41419-021-03618-7. 33771980 PMC7997956

[7] P.‐H. Liao , H.‐H. Hsu , T.‐S. Chen , et al., “Phosphorylation of Cofilin‐1 by ERK Confers HDAC Inhibitor Resistance in Hepatocellular Carcinoma Cells via Decreased ROS‐Mediated Mitochondria Injury,” Oncogene 36, no. 14 (2017): 1978–1990, 10.1038/onc.2016.357. 27748761

[8] H. Y. Choi , J.‐H. Lee , K. H. Jegal , I. J. Cho , Y. W. Kim , and S. C. Kim , “Oxyresveratrol Abrogates Oxidative Stress by Activating ERK–Nrf2 Pathway in the Liver,” Chemico‐Biological Interactions 245 (2016): 110–121, 10.1016/j.cbi.2015.06.024. 26102008

[9] R. Checker , H. N. Bhilwade , S. R. Nandha , R. S. Patwardhan , D. Sharma , and S. K. Sandur , “Withaferin A, a Steroidal Lactone, Selectively Protects Normal Lymphocytes Against Ionizing Radiation Induced Apoptosis and Genotoxicity via Activation of ERK/Nrf‐2/HO‐1 Axis,” Toxicology and Applied Pharmacology 461 (2023): 116389, 10.1016/j.taap.2023.116389. 36716864

[10] Y. Zhang , F. Dong , Z. Cao , et al., “Eupalinolide A Induces Autophagy via the ROS/ERK Signaling Pathway in Hepatocellular Carcinoma Cells In Vitro and In Vivo,” International Journal of Oncology 61, no. 5 (2022): 131, 10.3892/ijo.2022.5421. 36111510 PMC9507091

[11] Z. Yuan , Z. Liang , J. Yi , et al., “Koumine Promotes ROS Production to Suppress Hepatocellular Carcinoma Cell Proliferation via NF‐κB and ERK/p38 MAPK Signaling,” Biomolecules 9, no. 10 (2019): 559, 10.3390/biom9100559. 31581704 PMC6843837

[12] D. Wang , H. Xu , L. Fan , et al., “Hyperphosphorylation of EGFR/ERK Signaling Facilitates Long‐Term Arsenite‐Induced Hepatocytes Epithelial‐Mesenchymal Transition and Liver Fibrosis in Sprague‐Dawley Rats,” Ecotoxicology and Environmental Safety 249 (2023): 114386, 10.1016/j.ecoenv.2022.114386. 36508792

[13] D. Savic , T. B. Steinbichler , J. Ingruber , et al., “Erk1/2‐Dependent HNSCC Cell Susceptibility to Erastin‐Induced Ferroptosis,” Cells 12, no. 2 (2023): 336, 10.3390/cells12020336. 36672272 PMC9856753

[14] N. Liu , Y. Liang , T. Wei , et al., “The Role of Ferroptosis Mediated by NRF2/ERK‐Regulated Ferritinophagy in CdTe QDs‐Induced Inflammation in Macrophage,” Journal of Hazardous Materials 436 (2022): 129043, 10.1016/j.jhazmat.2022.129043. 35525219

[15] W.‐T. Chang , Y.‐D. Bow , P.‐J. Fu , et al., “A Marine Terpenoid, Heteronemin, Induces Both the Apoptosis and Ferroptosis of Hepatocellular Carcinoma Cells and Involves the ROS and MAPK Pathways,” Oxidative Medicine and Cellular Longevity 2021, no. 1 (2021): 7689045, 10.1155/2021/7689045. 33488943 PMC7803406

[16] D. Huang , X. Dong , J. Li , et al., “Steroidal Saponin SSPH I Induces Ferroptosis in HepG2 Cells via Regulating Iron Metabolism,” Medical Oncology 40, no. 5 (2023): 132, 10.1007/s12032-023-02000-1. 36977862

[17] J.‐l. Zhou , X.‐y. Huang , H.‐c. Qiu , et al., “SSPH I, a Novel Anti‐Cancer Saponin, Inhibits Autophagy and Induces Apoptosis via ROS Accumulation and ERK1/2 Signaling Pathway in Hepatocellular Carcinoma Cells,” Oncotargets and Therapy 13 (2020): 5979–5991, 10.2147/ott.S253234. 32606806 PMC7320904

[18] G. Liang , Y. Sun , M. Ou , B. Liu , H. Qiu , and Y. Chen , “A Method for the Isolation of a Saponin Compound,” China. CN201510436677.4 (2017).

[19] M. Ou , “Effects of Saponins from Schizocapsa Plantaginea Hance on Apoptosis and Cell Cycle Regulation in Hepatocellular Carcinoma Cells and Purification of the Saponins,” (Nanning, Guangxi, China: Guangxi Medical University, 2015).

[20] S. Hu , J. Feng , M. Wang , et al., “Nrf1 Is an Indispensable Redox‐Determining Factor for Mitochondrial Homeostasis by Integrating Multi‐Hierarchical Regulatory Networks,” Redox Biology 57 (2022): 102470, 10.1016/j.redox.2022.102470. 36174386 PMC9520269

[21] G. Miotto , M. Rossetto , M. L. Di Paolo , et al., “Insight Into the Mechanism of Ferroptosis Inhibition by Ferrostatin‐1,” Redox Biology 28 (2020): 101328, 10.1016/j.redox.2019.101328. 31574461 PMC6812032

[22] X. Zhang , M. Zhang , Z. Zhang , and S. Zhou , “Salidroside Induces Mitochondrial Dysfunction and Ferroptosis to Inhibit Melanoma Progression Through Reactive Oxygen Species Production,” Experimental Cell Research 438, no. 1 (2024): 114034, 10.1016/j.yexcr.2024.114034. 38588875

[23] Y. Liu , X. Wang , C.‐Y. Sun , and J. Wang , “Delivery of Mitogen‐Activated Protein Kinase Inhibitor for Hepatocellular Carcinoma Stem Cell Therapy,” ACS Applied Materials & Interfaces 7, no. 1 (2014): 1012–1020, 10.1021/am508262j. 25522342

[24] P. J. Klein , C. M. Schmidt , C. A. Wiesenauer , et al., “The Effects of a Novel MEK Inhibitor PD184161 on MEK‐ERK Signaling and Growth in Human Liver Cancer,” Neoplasia 8, no. 1 (2006): 1–8, 10.1593/neo.05373. 16533420 PMC1601146

[25] J. Zhou , J. Luo , R. Gan , et al., “SSPH I, A Novel Anti‐Cancer Saponin, Inhibits EMT and Invasion and Migration of NSCLC by Suppressing MAPK/ERK1/2 and PI3K/AKT/mTOR Signaling Pathways,” Recent Patents on Anti‐Cancer Drug Discovery 19, no. 4 (2024): 543–555, 10.2174/0115748928283132240103073039. 38305308

[26] T. Wang , Z. Zhang , Z. Deng , et al., “Mesenchymal Stem Cells Alleviate Sepsis‐Induced Acute Lung Injury by Blocking Neutrophil Extracellular Traps Formation and Inhibiting Ferroptosis in Rats,” PeerJ 12 (2024): e16748, 10.7717/peerj.16748. 38304189 PMC10832623

[27] M. Rebollo‐Hernanz , Y. Aguilera , M. A. Martin‐Cabrejas , and E. Gonzalez de Mejia , “Phytochemicals From the Cocoa Shell Modulate Mitochondrial Function, Lipid and Glucose Metabolism in Hepatocytes via Activation of FGF21/ERK, AKT, and mTOR Pathways,” Antioxidants 11, no. 1 (2022): 136, 10.3390/antiox11010136. 35052640 PMC8772970

[28] Y. Luo , J. Ma , and W. Lu , “The Significance of Mitochondrial Dysfunction in Cancer,” International Journal of Molecular Sciences 21, no. 16 (2020): 5598, 10.3390/ijms21165598. 32764295 PMC7460667

[29] H.‐Y. Lee , H. T. Nga , J. Tian , and H.‐S. Yi , “Mitochondrial Metabolic Signatures in Hepatocellular Carcinoma,” Cells 10, no. 8 (2021): 1901, 10.3390/cells10081901. 34440674 PMC8391498

[30] Y. Q. Zhang , K. N. Li , J. H. Cui , Y. F. Liu , and S. J. Yang , “Abnormal Expression of NRF‐2α in Hepatocellular Carcinoma Identified With a Newly Prepared Monoclonal Antibody Against Human NRF‐2α Protein,” Molecular Biology Reports 38, no. 5 (2010): 3083–3088, 10.1007/s11033-010-9976-6. 20127517

[31] Y. Sun , “Anti‐HCC Study of Saponins from Schizocapsa Plantaginea (Hance) and Saponin Compound SSPH Inhibite Hela Cell Proliferation by Regulating ROS‐ERK Signal,” (China, Guangxi: Guangxi Medical University, 2016).

[32] Y. Tao , W. Zhou , C. Chen , et al., “O‐Sialoglycoprotein Endopeptidase (OSGEP) Suppresses Hepatic Ischemia‐Reperfusion Injury‐Induced Ferroptosis Through Modulating the MEK/ERK Signaling Pathway,” Molecular Biotechnology 67, no. 2 (2025): 689–704, 10.1007/s12033-024-01084-y. 38456959 PMC11711258

[33] C. Zhao , Y. Yu , G. Yin , et al., “Sulfasalazine Promotes Ferroptosis Through AKT‐ERK1/2 and P53‐SLC7A11 in Rheumatoid Arthritis,” Inflammopharmacology 32, no. 2 (2024): 1277–1294, 10.1007/s10787-024-01439-6. 38407703 PMC11006818

[34] Q. Xiong , X. Tian , C. Xu , et al., “PM2.5 Exposure‐Induced Ferroptosis in Neuronal Cells via Inhibiting ERK/CREB Pathway,” Environmental Toxicology 37, no. 9 (2022): 2201–2213, 10.1002/tox.23586. 35608139

[35] K. Du , S. H. Oh , R. K. Dutta , et al., “Inhibiting xCT/SLC7A11 Induces Ferroptosis of Myofibroblastic Hepatic Stellate Cells but Exacerbates Chronic Liver Injury,” Liver International 41, no. 9 (2021): 2214–2227, 10.1111/liv.14945. 33991158 PMC8594404

[36] N. Diniyah , M. B. Alam , H.‐J. Choi , and S.‐H. Lee , “ *Lablab purpureus* Protects HaCaT Cells From Oxidative Stress‐Induced Cell Death Through Nrf2‐Mediated Heme Oxygenase‐1 Expression via the Activation of p38 and ERK1/2,” International Journal of Molecular Sciences 21, no. 22 (2020): 8583, 10.3390/ijms21228583. 33202535 PMC7697790

[37] Y. Yang , X. Wei , M. Ying , et al., “Natural Pyrethrin‐Induced Oxidative Damage in Human Liver Cells Through Nrf‐2 Signaling Pathway,” Toxics 12, no. 4 (2024): 258, 10.3390/toxics12040258. 38668481 PMC11053901

[38] J. Singh , A. Phogat , V. Kumar , and V. Malik , “N‐Acetylcysteine Ameliorates Monocrotophos Exposure‐Induced Mitochondrial Dysfunctions in Rat Liver,” Toxicology Mechanisms and Methods 32, no. 9 (2022): 686–694, 10.1080/15376516.2022.2064258. 35403558

[39] X. Zhang , Y. Yu , H. Lei , et al., “The Nrf‐2/HO‐1 Signaling Axis: A Ray of Hope in Cardiovascular Diseases,” Cardiology Research and Practice 2020 (2020): 1–9, 10.1155/2020/5695723. PMC7204387 32411446

[40] R. R. Ratan , “The Chemical Biology of Ferroptosis in the Central Nervous System,” Cell Chemical Biology 27, no. 5 (2020): 479–498, 10.1016/j.chembiol.2020.03.007. 32243811 PMC7245561

[41] Y. Zhang , Y. Qu , R. Cai , et al., “Atorvastatin Ameliorates Diabetic Nephropathy Through Inhibiting Oxidative Stress and Ferroptosis Signaling,” European Journal of Pharmacology 976 (2024): 176699, 10.1016/j.ejphar.2024.176699. 38825302

[42] H. N. Mai , D. T. Pham , Y. H. Chung , et al., “Glutathione Peroxidase‐1 Knockout Potentiates Behavioral Sensitization Induced by Cocaine in Mice via Sigma‐1 Receptor‐Mediated ERK Signaling: A Comparison With the Case of Glutathione Peroxidase‐1 Overexpressing Transgenic Mice,” Brain Research Bulletin 164 (2020): 107–120, 10.1016/j.brainresbull.2020.08.011. 32822804

[43] M. E. Abo‐El Fetoh , G. K. Helal , I. G. Saleh , et al., “Cyclosporin A Activates Human Hepatocellular Carcinoma (HepG2 Cells) Proliferation: Implication of EGFR‐Mediated ERK1/2 Signaling Pathway,” Naunyn‐Schmiedeberg's Archives of Pharmacology 393, no. 5 (2020): 897–908, 10.1007/s00210-019-01798-w. 31907582

[44] Y. Zhu , S. Wang , P. Niu , et al., “Raptor Couples mTORC1 and ERK1/2 Inhibition by Cardamonin With Oxidative Stress Induction in Ovarian Cancer Cells,” PeerJ 11 (2023): e15498, 10.7717/peerj.15498. 37304865 PMC10257395

[45] G. Zhang , J. He , X. Ye , et al., “Beta‐Thujaplicin Induces Autophagic Cell Death, Apoptosis, and Cell Cycle Arrest Through ROS‐Mediated Akt and p38/ERK MAPK Signaling in Human Hepatocellular Carcinoma,” Cell Death & Disease 10, no. 4 (2019): 255, 10.1038/s41419-019-1492-6. 30874538 PMC6420571

[46] G. Zhan , T. Wei , H. Xie , et al., “Autophagy Inhibition Mediated by Trillin Promotes Apoptosis in Hepatocellular Carcinoma Cells via Activation of mTOR/STAT3 Signaling,” Naunyn‐Schmiedeberg's Archives of Pharmacology 397, no. 3 (2024): 1575–1587, 10.1007/s00210-023-02700-5. 37676495

[47] C. Zhang , Y. Yu , Q. Huang , and K. Tang , “SIRT6 Regulates the Proliferation and Apoptosis of Hepatocellular Carcinoma via the ERK1/2 Signaling Pathway,” Molecular Medicine Reports 20, no. 2 (2019): 1575–1582, 10.3892/mmr.2019.10398. 31257493 PMC6625461

